# Perspectives on an enhanced ‘Improving Access to Psychological Therapies’ (IAPT) service addressing the wider determinants of mental health: a qualitative study

**DOI:** 10.1186/s12913-023-09405-8

**Published:** 2023-05-24

**Authors:** Esther Louise Curtin, Katrina d’Apice, Alice Porter, Emily Widnall, Matthew Franklin, Frank de Vocht, Judi Kidger

**Affiliations:** 1 Department of Population Health, Faculty of Epidemiology and Population Health, London School of Hygiene and Tropical Medicine, Keppel Street, London, WC1E 7HT UK; 2 Centre for Public Health, Population Health Sciences, Bristol Medical School, University of Bristol, Canynge Hall, 39 Whatley Road, Bristol, BS8 2PS UK; 3 School for Health and Related Research (ScHARR), University of Sheffield, Regent Court, Sheffield, S1 4DA UK; 4 NIHR Applied Research Collaboration West (NIHR ARC West), Whitefriars, Lewins Mead, Bristol, BS1 2NT UK

**Keywords:** Mental health services, Signposting, Health promotion, Stakeholder perspectives, Service user experience, Qualitative

## Abstract

**Background:**

A new Health and Wellbeing pathway was introduced into the Improving Access to Psychological Therapies (IAPT) service in one geographical area of the UK in 2021 to address the wider determinants of mental health problems. It comprised assisted signposting to wider services and physical health promotion. This qualitative study aimed to understand stakeholders’ experiences of implementing and receiving this new support and the barriers and facilitators to its delivery.

**Methods:**

Forty-seven interviews were conducted, with service developers (n = 6), service deliverers (n = 12), service users (n = 22) and community and clinical partners (n = 7), as part of a larger mixed-methods evaluation. Interviews were recorded, transcribed, and analysed using reflexive thematic analysis.

**Results:**

Three themes spanned all participant groups and represented key aspects of the service: (1) identifying suitability, (2) a holistic service, and (3) moving forward. The sub-themes represent the barriers and facilitators to processes working in practice, lending insight into potential service improvements. These included strengthening the quality of communication during referral and assessment, tailoring the support and delivery mode, and increasing transparency around continued care to drive sustained benefits.

**Limitations:**

Service users may have been selected due to their positive experiences of IAPT and were not demographically representative of the population, although participants’ experiences of the service did suggest variation in our sample.

**Conclusions:**

The Health and Wellbeing pathway was perceived as having a positive impact on mental health and could reduce the burden on therapeutic services. However, service- and individual-level barriers need to be addressed to enhance statutory and community support links, manage service users’ expectations, and improve accessibility for certain groups.

## Introduction

One in two people in the UK will experience a mental health condition in their lifetime [1], and mental health conditions are associated with a 20-year reduction in longevity [2]. A meta-analysis reported an increase in the prevalence of depression and anxiety exceeding 25% during the COVID-19 pandemic [3], and disproportionate vulnerability in minority groups raises concerns around inequality [4]. Effective healthcare services are integral to tackling this crisis [5]. England’s national ‘Improving Access to Psychological Therapies’ (IAPT) service delivers talking therapy to over one million adults per year [6], and is recognised by policymakers and clinicians as conferring population-level benefits [7]. However, evidence suggests effectiveness is limited to the short-term [8, 9], possibly due to failure to address the wider determinants of mental health across social, environmental and lifestyle domains [10]. Moreover, those accessing IAPT lack diversity, with service users predominately 18-35-years-old (54.6%), female (66.8%), and White (74.0%) [6]. Thus, achieving an equitably accessed service that leads to sustained benefits is a major priority.

In recent years, there has been a policy shift for the treatment of common mental health problems from solely offering therapeutic and medical treatment towards an interdisciplinary approach where services deliver ‘direct interventions’ (in-house support) and ‘connector interventions’ (integrating statutory and voluntary services) [11]. Examples include interventions focused on debt advice, befriending, physical activity, and dietary improvement, as well as social prescribing or ‘signposting’, whereby a health professional advises service users to access wider community support [1, 12]. Reviews of such approaches document possible mechanisms of change including reduced stress, social isolation and rumination [13], but indicate stronger evidence exists for improvement during and immediately post-intervention while longer-term benefits are less clear [14, 15]. Signposting shows promise for reducing health inequalities, as signposting service users to informal support, delivered by community workers as opposed to health professionals, may be considered more accessible, approachable and less stigmatising, and may lead to increasing patient autonomy and empowerment to then access more formal services if required [14, 15]. Despite the compelling evidence, implementation into practice remains slow, which may be explained by barriers at the service and service user level [16]. Moreover, there remains a paucity of high-quality process evaluations on services that offer both health promotion and signposting alongside traditional talking therapy.

While previous studies have explored how IAPT links with specific support, for example, employment services [17], research has yet to be conducted on linking to a wider array of community services, and the impact of a bespoke workforce forging these links. This paper presents findings from a qualitative study, part of a larger mixed-methods evaluation of a new delivery model for IAPT introduced into one area of England, which builds on features of signposting and health promotion to address the wider determinants of mental health issues [18, 19]. The Medical Research Council’s new framework calls for comprehensive process evaluations of complex interventions such as these, integrating stakeholder perspectives to capture feasibility and acceptability, and inform future transformation and replication [20]. Our qualitative study aimed to explore how the new Health and Wellbeing Pathway within IAPT was delivered and received, and the barriers and facilitators to implementation and impact from the perspectives of service developers, deliverers, users, and clinical and community partners.

## Methods

### Setting

This study examined a new delivery model for the IAPT service implemented in April 2021 by one local commissioning body in one geographical area in England.

**Fig. 1:**
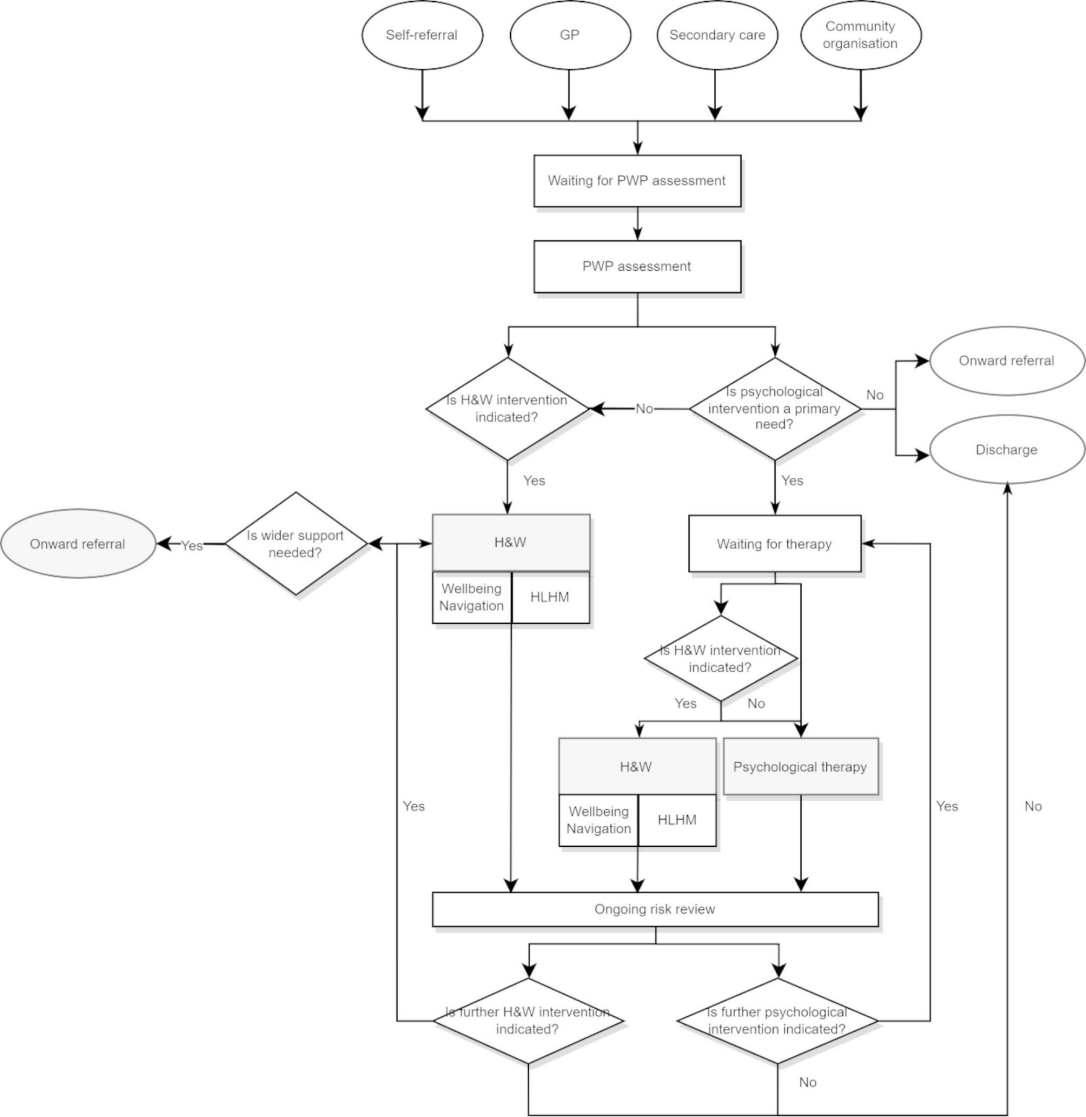
Visual Representation of the Enhanced Improving Access to Psychological Therapies Service Structure Grey shapes indicate the four types of support offered after the PWP assessment (‘stepped-care’ psychological treatment, onward referral, Health and Wellbeing pathway while awaiting therapy, or Health and Wellbeing pathway only); rectangle boxes indicate activities within IAPT; circles indicate activities outside of IAPT; diamonds indicate ongoing review questions Key terms: GP – General Practitioner. PWP – Psychological Wellbeing Practitioner. H&W – Health and Wellbeing Pathway; HLHM – Healthy Living Healthy Minds programme

The core IAPT service provides NICE-approved cognitive behaviour therapy (CBT) to service users with common mental health problems, which is delivered via telephone, online, or in-person [21]. The IAPT service also offers counselling for depression and interpersonal therapy, however on a smaller scale than CBT. In the traditional model, after being referred into IAPT (often through a GP or a self-referral), service users undergo an assessment by a Psychological Wellbeing Practitioner (PWP) and are offered two options: ‘stepped-care’ psychological treatment whereby those with mild to moderate symptoms are first offered low intensity CBT, and then high intensity CBT if required, or signposting advice/onward referral, e.g., to high-intensity treatment or a community service. In the new model, service users still undergo the PWP needs assessment, but then are offered two additional options: the Health and Wellbeing pathway only (Wellbeing Navigation or the Healthy Living Healthy Minds (HLHM) programme), or the Health and Wellbeing pathway while waiting for therapy. Typically, service users are offered one of either Wellbeing Navigation or HLHM as they seek to address distinct needs and issues, but they can be offered the other later in their treatment course if needed. See Fig. 1 for further details of the treatment pathways.

#### Wellbeing navigation

Service users receive one-to-one sessions with a Wellbeing Navigator to facilitate connections with community organisations that can address practical problems including poverty, unemployment, and social isolation. Wellbeing Navigation includes an initial review call to establish needs, lasting 45 min, and up to six further calls, lasting 30 min each, which are spaced either one to two weeks apart depending on the level of support required and the service user’s availability.

#### ‘Healthy Living Healthy Minds’ programme (HLHM)

Service users attend group sessions in the form of six group webinars targeting behaviour change to achieve a healthy lifestyle, through informative presentations and guided exercise classes delivered by ‘Health and Wellbeing Coaches’. Similar to Wellbeing Navigation, the support begins with a review call, this time lasting one hour, and subsequent 30-minute one-to-one calls every one to two weeks alongside the group sessions, and the entire programme typically lasts 12 weeks.

The employees recruited as Wellbeing Navigators came from a range of backgrounds but often had previous experience of social prescribing to support people with mental health difficulties. To be recruited as a Health and Wellbeing Coach, applicants must have completed a minimum of Level 3 qualification in exercise instruction (e.g., Level 3 Personal Training), which covers the technical content. The training for both Wellbeing Navigators and Health and Wellbeing Coaches involves service-specific training on screening for risk, writing notes, familiarisation with the patient system, using the database of local services to refer into, shadowing calls and practicing role plays of real-life scenarios. Wellbeing Navigators also received presentations from external speakers updating them on their community offering.

Due to the COVID-19 pandemic, all IAPT support delivered during this study was delivered remotely (Wellbeing Navigation via telephone, HLHM via telephone and video call). Community services offered a mix of face-to-face and telephone support.

### Study design and recruitment

The Assessing a Distinct Improving Access to Psychological Therapies service (ADAPT) study was a mixed methods two-centre non-randomised intervention study that evaluated the effectiveness, cost-effectiveness, and acceptability of IAPT’s new Health and Wellbeing pathway, and the protocol is published elsewhere [22]. For the qualitative component of the ADAPT study, we sought to identify the views of stakeholders from four groups: (1) individuals involved in the specification and development of the new pathway (‘service developers’), (2) practitioners delivering the service (‘service deliverers’), (3) staff from external community or clinical organisations that receive referrals from or refer into IAPT (‘partners’), and (4) service users who previously or currently received IAPT support, either therapy only, the Health and Wellbeing pathway, or both (‘service users’).

The study team identified potential participants from the former three groups through consulting the IAPT service managers and subsequent snowball sampling. We aimed to yield a broad enough group of stakeholders who could comment on different aspects of the service’s implementation and delivery (e.g., service commissioners, managers, leads, and practitioners). For service users, we first asked the IAPT patient liaison officer, who had access to service users’ email addresses due to their communication and administrative responsibilities, to send a broadcast email to all service users who had been discharged. However, as this mainly yielded therapy-only service users, we asked the service deliverers themselves to individually approach service users in the treatment sessions and prioritise individuals from under-represented populations, including older adults and ethnic minority groups. This was an attempt to maximise diversity to engage service users regardless of how long they had been in the service and the outcomes they had experienced. Contact details of those who expressed an interest and gave permission to be contacted were shared with the study team and they were invited for interview, providing they met the following eligibility criteria: (1) aged 18 or over; (2) sufficiently fluent in written and spoken English; and (3) able to give informed consent, written or spoken. To aid this process, one of the researchers visited a Wellbeing Navigator team meeting to discuss the importance of recruiting a diverse group of service users in order for our findings to be impactful for the maximum number of users, e.g., those from more deprived backgrounds and who experienced issues with the service. We felt that by emphasising the aims of the study to service deliverers, this increased the diversity and relevance of views represented, and thus the information power in relation to answering our research objectives [23]. While therapy-only service users could not comment on the new pathway, we felt it appropriate to include these participants to explore the contrast in experiences.

The study was performed in accordance with the Declaration of Helsinki. Ethical approval was granted by the NHS Research Ethics Committee (Reference number: 21/PR/0230). All participants provided informed consent prior to the interviews taking place.

### Participants

Forty-seven interviews were conducted with service developers (n = 6), service deliverers (n = 12), partners (n = 7), and service users (n = 22) (Table 1). The 15 Health and Wellbeing pathway service users were aged between 28 and 73-years-old (mean age 46.5 years, 71.4% between 36 and 64-years- old, and 6.7% between 18 and 25-years-old), the majority identified as male (61.5%), and they were mostly White British (92.9%). By comparison, national IAPT data for referrals between April 2020 and April 2021 show service users were slightly younger (54.3% aged between 18 and 35-years-old), and mostly female (67.2%), but the majority also identified as White (74.2%) [24]. We are unaware of how many service users declined to participate in interviews when initially approached by the service deliverer, but out of the 38 who agreed to be contacted by the study team, 14 did not respond, and two chose not to participate after reading the information sheet.

**Table 1:** Details of Participant Group and Role and/or Involvement in the Service

Participant group	Role and/or involvement		Total	
Service developers	Commissioners, Local authority Public Health Managers, IAPT Service Managers, public advisors		6	
Service deliverers	Clinical Leads, PWPs, Health and Wellbeing Service Leads, Wellbeing Navigators, Health and Wellbeing Coaches		12	
Community and clinical partners^1^	Chief Executives, Managers, Counsellors, General Practitioners		7	
Service users			22	
	IAPT therapy-only (therapy before the Health and Wellbeing pathway was introduced)		7	
	Health and Wellbeing pathway^2^			
		Wellbeing Navigation	8	
		HLHM	7	
Total			47	

^1^Services providing nature-based therapy, perinatal care, befriending, peer-support, and counselling

^2^Of these 15 service users, 3 received therapy before, 5 were awaiting therapy, and 7 were not expecting to receive therapy

Key terms: PWP = Psychological Wellbeing Practitioner; IAPT = Improving Access to Psychological Therapies; HLHM = Healthy Living Healthy Minds

### Procedure

Semi-structured interviews were conducted between May and December 2021. The duration of the interviews ranged from 14 to 59 min, and they were mainly conducted via telephone, with one being face-to-face, and one via video call. The topic guides were tailored for each group to explore their experiences of the new pathway, and the interview questions were discussed with two public advisors to ensure comprehensibility and relevance. A summary of the topics covered is displayed in Table 2. Demographic information (age, gender, and ethnicity) was collected from Health and Wellbeing service users. All participants provided informed consent prior to commencing the interviews and all service users received a £20 voucher as acknowledgement of their time.

**Table 2:** Summary of Interview Topic Guides for Staff and Service Users

Staff^1^	Service user
Can you describe your role in relation to IAPT?	Can you walk me through how you accessed IAPT?
How does the Health and Wellbeing pathway support service users?	What was the assessment process like at the beginning?
How does the pathway link service users with additional services?	How did you find the support you received?
What are the issues stopping the new pathway from addressing wider causes of mental health?	Was the support you received what you expected?
What are facilitators helping the new pathway address wider causes of mental health?	How could your experience be improved?
What improvements could be made to the Health and Wellbeing pathway?	Were you offered anything extra during or after your support?

^1^Included service developers, service deliverers, and clinical and community partners

Key terms: IAPT = Improving Access to Psychological Therapies

### Analysis

The interview audio recordings were transcribed verbatim, anonymised, and imported into NVivo (Release 1.6.1). Audio recordings and transcripts were uploaded and stored on a secure university network drive, only accessible to members of the research team. Data were analysed inductively following Braun and Clarke’s reflexive thematic analysis approach, which posits that there is not one meaning to be uncovered through consensus of two or more coders, but rather meaning is generated by the individual doing the analysis. Our validity checks came from team discussions of the themes, and checking that the final themes reflected our codes and the original data [25]. No existing theoretical frameworks were drawn upon during the analysis due to the novelty of the research question in relation to previous work. Two researchers (ELC and KD) read, re-read and annotated the transcripts, and ELC generated the initial codes. ELC, KD and a third researcher, JK, discussed the codes and identified potential emerging themes for each participant group separately. ELC compared the themes across the four groups to create one list of themes. Following this, ELC presented the candidate themes at an advisory group meeting with the wider ADAPT study team including our public advisors, to refine the themes and theme names. The main change that came out of the discussions was that the themes were initially named to reflect the different points on the treatment pathway, i.e., ‘Initial referral’, ‘Treatment’, and ‘Follow-up’, but these were perceived as not being very informative or interpretive, and therefore the final themes were reworked to encapsulate the key messages arising from participants’ accounts about where the service did or did not work well. To confirm accuracy, ELC returned to the data multiple times throughout the process to re-read the transcripts and confirm the themes reflected the content of the dataset as a whole.

Adhering to a constructionist epistemology, the researchers used subjectivity to interpret meaning in the accounts [25]. ELC (MSc) and KD (PhD) are Public Health Researchers and JK (PhD) is a Senior Lecturer in Public Health specialising in mental health interventions. ELC, KD and JK have extensive experience in qualitative research across a range of public health studies and all remained conscious of their backgrounds and positions in light of the topic in order to minimise the risk of bias during the analytical process. The main researcher, ELC, is a White female early career researcher in her mid-20s with no personal experience of accessing mental health services. Due to ELC conducting most of the interviews and therefore having a good understanding of the context and meaning in what was discussed, the analysis naturally transitioned from semantic to latent coding, from merely describing the content to generating more implicit, ‘hidden’ meaning underneath the surface-level summaries [26].

## Results

**Fig. 2:**
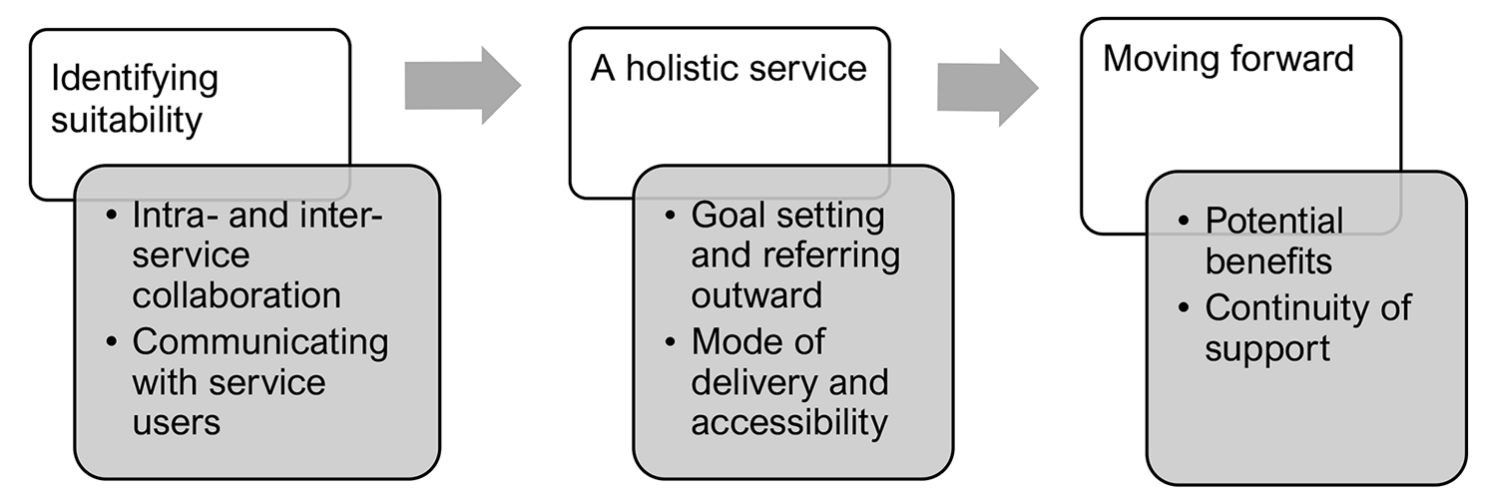
Main Themes and Subthemes

We present three key themes that spanned across all participant groups, relating to key ways the service is perceived to work: (1) identifying suitability, (2) a holistic service, and (3) moving forward. Each theme was made up of two subthemes, which represented recurring facilitators or barriers to the effectiveness of that component of the service and affected whether it materialised as a benefit or shortcoming in a particular context. Figure 2 provides a visual representation of the themes and subthemes.

### Theme 1: identifying suitability

Many stakeholders discussed the process of the initial referral into IAPT, and the PWP assessment that sought to identify whether the service user’s needs could be met by the pathways operating in-house or whether they would benefit from external support. Ascertaining suitable and available support in the community was key to the Wellbeing Navigator’s role, and service users generally reported that their interactions with Wellbeing Navigators made the process of finding suitable support easier. The strength of collaboration amongst service deliverers, and between service deliverers and users, impacted the success of this suitability identification process.

#### Intra- and inter-service collaboration

Service deliverers and community partners expressed mixed feelings regarding how well the suitability identification process worked in practice. The occasional occurrence of inappropriate referrals from the PWP assessment to the Health and Wellbeing pathway and from the Health and Wellbeing pathway to community organisations was attributed to limitations in communication and/or a lack of familiarity with the respective services. PWPs’ understanding of the role of the Health and Wellbeing pathway was necessary to ensure appropriate referrals. Therefore, to facilitate understanding of the new pathway and integrate PWPs into the new model, PWPs participated in a HLHM ‘pilot’ programme to experience it from the service users’ perspective. In addition, service managers explained the Health and Wellbeing pathway during team meetings, and PWPs asked service users for feedback early on. This helped PWPs to view the new service more positively and overcome a sense of change fatigue that accompanied organisational changes.

*“[Service managers] came to our clinical skills groups that we have every week … talked through what the pathway was, who was suitable and how to refer … there just seems to be always a lot of change, and some feedback from clinicians was: ‘It’s hard to keep up.’ But I think, generally speaking, it’s been really good” (Service Deliverer 8, PWP).*

In terms of inter-service collaboration, Wellbeing Navigators encountered difficulties discerning appropriate wider services to connect service users with, due to a lack of named contacts when seeking further information about available support services. Furthermore, plans to co-locate with other services (practitioners with varied expertise being based in the same location) did not come to fruition, partly due to COVID-19-related restrictions.

*“The gold standard would be for some of us to be much more co-located ... a team of people in there that might include a psychologist, some doctors, some social prescribers, a mental health nurse, someone from IAPT ... you can just discuss the person’s needs and get them to the right place” (Service Developer 2, IAPT Service Manager).*

In addition, community organisations having limited capacity and complicated eligibility criteria reduced the Wellbeing Navigators’ inclination to connect service users with them. For example, *Service Deliverer 7 (Wellbeing Navigation)* described services only being accessible to *“certain postcodes … dependent on where their GP is based”*, but this was not clearly laid out on their websites, and other participants mentioned a need for more timely updating of service changes to avoid redundant referrals.

*“One patient the other day that had been referred by Wellbeing Navigation ... but the service has since closed ... keeping our intranet up to date ... maybe link up with other people’s databases” (Service Deliverer 1, HLHM).*

#### Communication with service users

Even when the PWPs understood the value of the Health and Wellbeing pathway, there were still concerns regarding whether the initial assessment enabled them to identify wider needs to then pinpoint appropriate routes of support. The primary purpose of the assessment was to evaluate psychological wellbeing and determine the need for therapy. Depending on the severity of the service user’s situation and the PWP’s caseload that day, the lifestyle questions could be missed all together. This was an ongoing challenge, as extending longer than an hour would not be effective as *“people get tired and then they might not take that information in” (Service Deliverer 9, PWP).* Giving service users more information and allowing online self-referrals directly into the Health and Wellbeing pathway was proposed to streamline the process. However, this relied on digital access and the service user having the confidence to ask for help.

*“I really like the HLHM video, so if there was a platform where you could find out about all these services, like ‘yep, I’m interested’ and that’s the referral. So that’s less work for the PWP, probably a more informed decision from the patient” (Service Deliverer 9, PWP).*

Once a service user was referred to them, staff within the Health and Wellbeing pathway made a further assessment and gathered information on their circumstances, knowledge, and readiness to change. This step was integral in delineating how they could actually support the service user and led to Wellbeing Navigators then compiling a list of relevant resources including charity helplines and websites to suit their needs.

*“They will have an assessment with a PWP, and from there they could be referred to us. There’s different types of criteria that they, not necessarily have to meet, because when we do speak to them, sometimes the criteria changes ... they may say one thing but actually it’s something else” (Service Deliverer 5, Wellbeing Navigation).*

Service users’ responses to being offered the Health and Wellbeing pathway were influenced by their expectations about the support they would receive, and how the new support was communicated to them at the point of referral and assessment. Those without clear expectations were generally more accepting of the non-clinical support, whereas those who expected therapy expressed more resistance. For example, *Service User 11 (Wellbeing Navigation)* came into IAPT expecting a less costly version of counselling, but after being offered Wellbeing Navigation while on the waitlist for therapy, they lost faith in the whole service and refused therapy when made available.

*“You can see on the GP notes, they said to the GP I just want to talk to someone about this, I really need some counselling ... that’s not really what we do ... we do try and say [we’re not a counselling service] in assessment, but after an hour of talking about all the most horrible bits of your life they might not be in a great place to really receive that” (Service Deliverer 12, Clinical Lead).*

*“I was hoping for some real support ... in the first instance it was, well it seems to be it functions like a signposting agency really and I didn’t get a lot from it” (Service User 11, Wellbeing Navigation).*

Both service users and deliverers emphasised the importance of receiving the right support at the right time, with some service users likely benefiting from having therapy first to address the symptoms of their mental health issue, while others needed to address practical issues beforehand otherwise *“that sense of helplessness might continue if they’re thinking there is no solution” (Service Provider 7, Wellbeing Navigation).* This was alluded to by *Service User 18* who disengaged from IAPT therapy due to their therapist not tackling wider issues occurring in their life.

*“I slipped through the net … if you’re slipping through the net because your mental health is getting worse because new things have happened, but they’re still desperately wanting help – I didn’t feel there was any option to say, ‘actually, life has just got a bit worse’” (Service User 18, IAPT therapy-only).*

### Theme 2: a holistic service

The principle aim of the Health and Wellbeing pathway was to provide a more holistic style of treatment. By linking IAPT with wider services in the community, the new pathway widened the access to groups for service users who would not necessarily engage with or adhere to treatment that is primarily focused on cognitive behavioural therapy. In this way, *Service Developer 5 (Local Authority Public Health Manager)* alluded to its direct relevance for people’s lives: *“I never thought of it as an enhanced service, more a logical service. So rather than adding something, one that turned the prior IAPT into its proper context”*. Setting personalised goals with service users and connecting outward were seen as enabling factors, while remote delivery and accessibility acted as either barriers or enablers depending on the circumstance. This theme highlights the contrasting experiences when service users received support from Wellbeing Navigators and Health and Wellbeing Coaches, with the latter seeming restricted to service users with internet access and more acceptable to those with lower levels of depression and anxiety that could be managed with lifestyle behaviour change and moderate signposting to wider services.

#### Goal setting and connecting outward

Following the referral into the Health and Wellbeing pathway, service users were contacted by phone whereby a practitioner further explored their situation and needs. This prevented service users feeling overwhelmed by all the different options and helped them to agree a *“clear and concise plan” (Service Deliverer 5, Wellbeing Navigation)*, and *“hone down” (Service Developer 2, IAPT Service Manager)* goals with their practitioner. Thereafter, service users perceived the regular structure of the sessions as helpful as it held them accountable to their goals.

*“He gave me goals and then every two weeks I had a conversation with him. They were booked in, so I knew that, throughout the week, if I wasn’t reaching my goal, he was going to ask” (Service User 13, HLHM).*

Both Wellbeing Navigators and Health and Wellbeing Coaches recommended wider services for specific problems. In HLHM, the onus remained on the service user to access these, and the coaches would only follow-up if “*it was to do with their lifestyle … to do with one of their goals” (Service Deliverer 1, Health and Wellbeing Coach).* By comparison, Wellbeing Navigators gave more attention to facilitating access, although the extent of their hands-on support varied. They did not typically make referrals on service users’ behalf, seeing their remit instead as increasing service users’ autonomy to access services or liaising with wider services to organise support from elsewhere. This meant that some service users still struggled to access services, with *Service Users 7 and 8* describing the cognitive effort required to *“pick up a phone and talk to someone*” and not having the *“guts”* to reach out.

*“I phoned [the Wellbeing Navigator] back to say, ‘would anybody come with me to the work capability assessment?’ and they said they did not, but that they would contact [the charity] Mind for me” (Service User 15, Wellbeing Navigation).*

Wellbeing Navigators’ support linking service users with external organisations reduced demand on PWPs to signpost during their assessment, such that they conducted *“a better assessment”*, and were able to spend more “*time prepping for your treatment sessions” (Service Deliverer 12, Clinical Lead).*

#### Mode of delivery and accessibility

The routine of the one-to-one sessions within the Health and Wellbeing pathway enabled rapport building and identification of coping strategies tailored to the individual, and many service users felt receiving support remotely facilitated a comfortable environment conducive to sharing personal issues. However, the video/online delivery of the HLHM programme seemed more appropriate for those who were digitally literate, whereas the Wellbeing Navigation support being delivered via telephone worked better for individuals who perhaps did not have consistent internet access and could not access community services in person. This sense of safety was also discussed in relation to the exercise sessions being delivered online benefitting those with body image concerns or social anxiety.

*“I’m sat in my little office at home, I feel secure in here. A meeting venue ... maybe I’ll feel a little bit vulnerable” (Service User 3, Wellbeing Navigation).*

Conversely, the quality of the HLHM educational webinars were seen as inferior online, which was echoed by service users from the standard service who felt the online CBT course hindered opportunity for interaction.

*“There were a few who sounded like they were just reading it off a board ... it’s very difficult when you, yourself, are just talking to a computer. You haven’t got any of that feedback” (Service User 21, IAPT therapy-only).*

The HLHM programme manager articulated plans to revert some of the sessions back to in-person after COVID-19. The remote online delivery was perceived as a barrier to reaching more marginalised groups including older adults and those not digitality literate. This mainly related to HLHM, because Wellbeing Navigation being delivered via telephone and offering “a *whole admin area that deals with translation” (Service Deliverer 5, Wellbeing Navigation)* was potentially more accessible to different groups. Nonetheless, certain communities were not reached, and a service manager suggested having a more diverse workforce could broaden the acceptability of the service.

*“Trying to diversify the workforce and get some people that really understand those communities ... I’d like to see someone from the asylum-seeker refugee community in the Wellbeing Navigation team, someone from the Muslim community” (Service Developer 2, IAPT Service Manager).*

Services in the community described having fewer access barriers, partly due to less stigma associated with seeking informal help from informal support compared to statutory support, and because they offered support targeted to different groups, and delivered in-person when possible, during the evolving COVID-19 restrictions.

*“We have our women of colour group, we have our LGBTQ + group, we have our women’s group, and we have our men’s group ... one member described it as being ‘shorthand’, that you don’t have to explain all the different challenges that you face” (Partner 1, Community Service Manager).*

### Theme 3: moving forward

Factors enabling the Health and Wellbeing Pathway to benefit service users in the long-term included the extent to which the support made an impact that was distinct from what they would get from therapy, and the ability to provide continued support beyond the time-bounds of the sessions.

#### Potential benefits

Positive impacts of the Health and Wellbeing Pathway included the mood-enhancing effects of exercise and the nurturing relationships that helped reduce feelings of social isolation.

*“A real person you can connect with … I live on my own with two children, so you know, getting cared for is something that had passed for me” (Service User 7, Wellbeing Navigation).*

Wellbeing Navigation was believed in some cases to have a *‘knock-on effect’ (Service Deliverer 6, Wellbeing Navigation)* on the family members of service users. After engaging in HLHM, service users did not always need their full course of therapy, meaning therapy could be reserved for those with more persistent issues.

*“They came for their online CBT, and they said, ‘I’d just like a few more techniques specifically around my mental health, but generally I’m feeling so much better’ ... half the work is done” (Service Deliverer 12, Clinical Lead).*

However, some service users described the uncertainty around being automatically discharged if they missed sessions, and whether that meant they remained on the therapy waitlist, as undermining the positive impacts they had gained thus far. While appreciating they could not be given a definitive time frame for the waitlist, they desired more communication about when they might expect the therapy.

#### Continuity of support

Staff from the Health and Wellbeing pathway described how the final treatment session focused on finding additional support for service users as most did need this, whether it be internal or external. Service users *“having open access” (Service User 13, HLHM)* to the Health and Wellbeing pathway if needed after the final session was deemed important, but was not uniformly known, which suggests some practitioners omitted this information or failed to communicate it effectively.

*“If I need further help, I’d have to go back to the doctor and get re-referred ... I’m not too sure how it works” (Service User 2, HLHM).*

If they wanted to re-refer themselves, some service users felt this was more efficient if it was done through a generic email address, whereas others preferred having the option to contact the same practitioner. Similarly, *Service User 11* sought more transparency when struggling to provide feedback following being discharged from Wellbeing Navigation.

*“All I got is people trying to ring me at various times, not leaving messages ... if I make a phone call, I don’t know who I’m speaking to ... it just gets lost in the system” (Service User 11, Wellbeing Navigation).*

Staff also discussed varied experiences of re-admission, with a Health and Wellbeing Coach explaining that they took it upon themselves to provide their personal contact details to allow direct contact, but they desired a more structured and streamlined way to do this, including a longer follow-up period and a smartphone app to aid communication.

*“It would be nice to be able to say, ‘oh, I’ll catch up with you in a month. We’ll do a three-month review’” (Service Deliverer 4, Health and Wellbeing Coach).*

## Discussion

This is the first study to evaluate a Health and Wellbeing pathway introduced into the IAPT service in England. Our three themes, (1) identifying suitability, (2) a holistic service, and (3) moving forward, reflect key pillars of the new treatment model from the perspectives of various stakeholders who implemented, delivered, and received aspects of the new support. Drawing on Normalisation Process Theory (NPT), a framework comprising four dimensions underpinning effective implementation of new practices [27], we highlight below the conditions needed for the pathway to be integrated and embedded into the routine IAPT service [28].

Our findings revealed inter-service communication and effective communication with service users as integral to identifying suitable treatment. This relates to three NPT dimensions: *coherence* amongst PWPs in understanding the role of the new pathway, leading to subsequent *cognitive participation* and *collective action*, expressed in their positive attitudes about offering the new service, and their engagement in training and building relationships with the new staff [27]. In contrast, the inadequacy of collaboration with community partners posed a barrier to Wellbeing Navigators linking with external organisations. Additionally, service users’ expectations for therapy, coupled with time constraints during assessment, meant they sometimes lacked clarity around the value of the new pathway. Therefore, barriers at the service- and individual-level affected the acceptability of being offered and offering the new support.

The breadth of the Health and Wellbeing pathway transformed IAPT into a more holistic service and provided service users with the opportunity to address wider determinants that may be impacting their mental health. This supports a review of IAPT research that highlighted the importance of expanding treatment options to serve the heterogeneous service user population [29]. We found the one-to-one sessions delivered remotely by the Wellbeing Navigators and Health and Wellbeing Coaches fostered tailoring and accountability, which aligns with research indicating remote delivery can widen accessibility to patients restricted by location, mobility, or transport, or with caring commitments [30]. On the other hand, the group sessions lacked interaction and resulted in amotivation. We do not theorise this to be a pitfall of group delivery versus one-to-one, but instead suggest it reflects the difficulty engaging in a large group and feeling a sense of social support through an online platform [31]. In accordance with our findings, previous studies exploring links between IAPT and employment and physical activity support emphasised the importance of continuity in the practitioner and the timeliness of advice [17, 32], and studies exploring experiences of therapy underlined sufficient time with the practitioner [33], and individualising advice to contexts such as the perinatal period [34]. Furthermore, while the remote delivery fostered notions of safety, our findings implied possible inequalities in access, which is reflected in the inverse care law, whereby those most in need often receive the least support [35], partly owing to a lack of confidence to seek help and possible digital exclusion [36].

Benefits of the Health and Wellbeing pathway included reduced stress and isolation, which at times led to reduced mental health symptoms and the need for therapy. However, this depended on the service users’ circumstances and expectations of receiving therapy initially, for example, when service users had milder anxiety or depressive symptoms and were more open to non-therapy routes, they were more likely to reap benefits from the new support compared to those with more severe symptoms. Additionally, service users relayed fears of being discharged prematurely if they missed sessions, and frustration with the sparse communication around future care. Encouragingly, aligning with NPT’s fourth construct, *reflexive monitoring* [27], the service deliverers engaged in formal and informal appraisal activities to assess the impact of the new support pathways. These were incorporated into specific changes, such that in December 2021, they introduced a review in the HLHM final session so service users could be removed from the therapy waitlist if deemed recovered, as well as text messages to request a reassessment in mid-waitlist. Both of these changes, which are clearly communicated upon referral, may relieve part of the uncertainty service users felt prior to this change.

### Strengths and limitations

This study is a novel exploration of delivering and receiving a mental health service that offers therapy and additional support for wider determinants, which provides transferable learning about successful service improvements. Our findings have highlighted challenges in implementing a joined-up service between statutory and voluntary mental health and wellbeing support and will be considered together with the quantitative results from the wider ADAPT research programme to understand whether the new model shows sufficient promise to warrant continuation and expansion, or if it requires substantial improvement first. These insights will have relevance to IAPT services across the country, who continually seek to improve patient outcomes. In addition, our findings could be transferred beyond IAPT and mental health services, as there is a general desire to create integrated partnerships between professional health services, public health prevention strategies, and non-health care organisations to achieve broad population health goals [37, 38].

We also note some study limitations. Selection bias may have resulted from relying on service deliverers to ask for expressions of interest from service users to take part in interviews, thus it was unclear whether service users were selected because of their positive views of IAPT. However, this was a practical decision we took due to the ethical challenges of requesting service users email addresses. We expect to be able to engage a larger, more diverse sample if a further evaluation was conducted, as recruitment was done in the early stages of the new service, when the number of referrals to the new pathway were still much lower than for therapy. Furthermore, participants expressed a range of positive and negative experiences, which suggests our sample was not highly selective or biased. Our final sample comprised participants who had followed different pathways and did include several service users who received therapy before the new pathway was introduced. While these participants had not directly received the new treatment, they provided comments on whether they would have accepted health and wellbeing support if it was offered to them, and we therefore decided to include their perspectives to explore potential contrasting experiences. To enable a clearer comparison of the perspectives of certain groups, it may have been helpful to present the demographic details from all study participants as well as the number of sessions and mental health diagnoses of service users, but we did not have this level of detail for each participant. As local IAPT services vary in their service users’ demographics, personnel structure, and resources, the local context needs to be considered if transferring our findings to other geographical areas and close stakeholder consultation could help to ensure acceptability.

## Conclusions

Our findings suggest it is possible to create a service addressing the wider determinants of mental health alongside clinical care, but acceptability and successful implementation relies on several factors. Firstly, communication across the system is necessary for service deliverers to understand the rationale for using a more holistic treatment style for primary prevention, and to effectively engage with this process. Secondly, the aims and eligibility criteria of community support need to be clear to facilitate the flow of referrals [17], as denoted in the UK Government’s plans for Integrated Care Partnerships [39] and the NHS’ long-term strategy for mental healthcare harnessing signposting [40]. Thirdly, modifying the assessment process to give service users more information about what the support entails rather than focusing only on needs and risk, could help establish trust and empathy from the start [41]. Fourthly, to enable an accessible, holistic service, service users could be given a choice of in-person or remote delivery, and digital education should be given accordingly [42, 43]. As identified previously, recruitment to diversify the workforce should be prioritised to ensure the relevance and perceived accessibility of support for under-represented populations such as ethnic minority groups [44]. Finally, keeping service users in the system post-discharge in case they require future support was highlighted as important. In conclusion, our study adds to the extant literature around mental health treatment, facilitating a deeper understanding of how interventions to address the wider determinants of mental health can be integrated into statutory care.

## Data Availability

The datasets generated and/or analysed during the current study are not publicly available due to the risk that the service may be identified due to its unique nature but are available from the corresponding author on reasonable request.

## Abbreviations

ADAPT: Assessing a Distinct Improving Access to Psychological Therapies service
HLHM: Healthy Living Healthy Minds
IAPT: Improving Access to Psychological Therapies
PWP: Psychological Wellbeing Practitioner
